# Individually Tailored Screening of Susceptibility to Sarcopenia Using p53 Codon 72 Polymorphism, Phenotypes, and Conventional Risk Factors

**DOI:** 10.1155/2014/743634

**Published:** 2014-10-13

**Authors:** Laura Di Renzo, Santo Gratteri, Francesca Sarlo, Andrea Cabibbo, Carmen Colica, Antonino De Lorenzo

**Affiliations:** ^1^Department of Biomedicine and Prevention, Division of Clinical Nutrition and Nutrigenomics, University of Rome “Tor Vergata”, Via Montpellier 1, 00133 Rome, Italy; ^2^Nuova Clinica Annunziatella, 00147 Roma, Italy; ^3^Department of Surgery and Medical Science, University “Magna Graecia”, 88100 Germaneto, Italy; ^4^Department of Agriculture, University of Naples “Federico II”, 80055 Portici, Italy; ^5^Department of Biology, University of Rome “Tor Vergata”, 00133 Rome, Italy; ^6^CNR, ISN UOS of Pharmacology, Department of Pharmacology, University “Magna Graecia”, 88100 Roccelletta di Borgia, Italy; ^7^National Institute for Mediterranean Diet and Nutrigenomics (I.N.Di.M.), 87032 Amantea, Italy

## Abstract

*Background and Aim.* p53 activity plays a role in muscle homeostasis and skeletal muscle differentiation; all pathways that lead to sarcopenia are related to p53 activities. We investigate the allelic frequency of the TP53 codon 72 in exon 4 polymorphism in the Italian female population and the association with appendicular skeletal muscle mass index in normal weight (NW), normal weight obese (NWO), and preobese-obese (Preob-Ob) subjects.* Methods.* We evaluated anthropometry, body composition, and p53 polymorphism in 140 women distinguished in NW, NWO, and Preob-Ob. Results. *Arg/*Arg genotype increases sarcopenia risk up to 20% (*Arg/*Arg genotype OR = 1.20; 95% CI = 0.48–2.9; *proallele carriers OR = 0.83; 95% CI = 0.83–2.06). The risk of being sarcopenic for *Arg/*Arg genotype in NWO and Preob-Ob is 31% higher than NW carriers of *proallele (RR = 0,31, 95% CI = 0,15–0,66, *P* = 0,0079). We developed a model able to predict sarcopenia risk based on age, body fat, and p53 polymorphism.* Conclusion*. Our study evidences that genotyping TP53 polymorphism could be a useful new genetic approach, in association with body composition evaluations, to assess sarcopenia risk.

## 1. Introduction

Interactions between genetic and environmental factors such as diet and lifestyle, particularly overnutrition and sedentary behavior, promote the progression and pathogenesis of polygenic diet-related diseases. Their current prevalence is increasing dramatically to epidemic proportions [1].

Certain single nucleotide polymorphisms (SNPs), occurring within the promoter region of a large number of genes, influence their transcriptional activity, resulting in intraindividual differences in the synthesis of coded proteins. A goal of the new postgenomic era is to uncover correlations between particular polymorphisms and the predisposition to develop certain diseases as a result of complex interaction of genetic, nutritional, and metabolic factors [2]. Once interesting genetic variants are identified, they can be used to screen the population for preventive medicine purposes.

In this context, the mounting influx of global quantitative data from both genetic, blood biomarkers and body composition, with a constant attention to disease and wellness, had led to a necessary transformation of the concept of medicine, intended not only as a curative intervention. It is necessary to transform healthcare to a proactive P4 medicine, that is* predictive*,* preventive*,* personalized*, and* participatory* [3]. This approach requires new strategies, both scientific and organizational, to enable bringing this revolution in medicine to patients and to the healthcare system. P4 medicine will have a profound impact on society, transforming the healthcare system, turning around the ever escalating costs of healthcare, digitizing the practice of medicine, and creating enormous economic opportunities for those organizations and nations that embrace this revolution [3]. To apply the P4 medicine system, new tools must be identified for facilitating relationships between scientists, care providers, patients, and consumers. The ability to sequence DNA and RNA fast is opening doors to many applications that could transform medicine, with a faster characterization of genetic disorders and identification of novel drug targets [4], and the possibility for earlier diagnoses.

Recent literature focuses attention on new roles for tumor protein p53 (TP53) beyond cancer progression, in processes such as metabolic syndrome, insulin resistance, adipose tissue inflammation, obesity, and skeletal muscle differentiation and homeostasis [5, 6].

In fact, in response to different genotoxic stimuli and depending on the degree of activation, p53 can promote transcriptional programs and protein-protein interactions which orchestrate DNA repair, cell cycle arrest, cellular stress responses, cell cycle regulation and differentiation [7], and senescence or apoptosis [8].

The gene,* TP53*, encoding p53, has a common sequence polymorphism in exon 4 of codon 72 characterized by a G to C substitution that determines the change of Arginine (Arg) to Proline (Pro) in the protein thereby coding for p53 variant R72P. The amino acidic change affects biochemical and functional properties of p53 protein. In fact, this polymorphism occurs in the Proline-rich domain of p53 protein, which is necessary for the protein to fully induce apoptosis. The Arg variant is a stronger apoptosis inducer, while the Pro variant is a stronger transcriptional activator [9].

Moreover, a putative role of TP53 was proposed in the severe and rapid skeletal muscle atrophy, which represents a hallmark of cachexia and of sarcopenia when the muscle atrophy occurs during aging [10]. However, although several studies reveal a role for TP53 during skeletal muscle differentiation and homeostasis, its precise mechanisms of action are still not well known, probably because they are complex and involve different metabolic pathways [11].

Various conditions leading to muscle wasting involve different pathways of intracellular signaling that trigger (i) programmed cell death (apoptosis); (ii) increased protein degradation through autophagy, calcium-dependent proteases (calpains and caspases), and proteasome system; (iii) decreased satellite cell activation, responsible for muscle regeneration [12]. Interestingly, all pathways that bring to sarcopenia are related to the activity of p53 gene, the so-defined “guardian of the genome” [13].

Sarcopenia is an “age-related” and “obesity-related” loss of muscle mass leading to muscle weakness, limited mobility, and increased susceptibility to injury; an understanding of the underlying causes of muscle loss is critical for the development of strategies and therapies to preserve muscle mass and function [14]. Sarcopenia has profound physiologic and clinical consequences, including but not limited to impaired protein turnover, mobility loss, osteoporosis, increased fracture risk, dyslipidemia, insulin resistance, overall frailty, and increased mortality. According to the European consensus on the definition and diagnosis of sarcopenia, this condition involves a loss of type II muscle fibers, a decline in total muscle area, the reduction of muscle capillarization, shortening velocity, and declining strength and/or physical performance. A necessary condition for a diagnosis of sarcopenia is therefore the extent of skeletal muscle mass loss that should be considered significant [15, 16]. Moreover, the combination of sarcopenia and obesity [17, 18], defined as sarcopenic obesity, is another important public health issue, associated with functional limitations and increased mortality.

Greater loss of muscle mass leading to sarcopenic obesity in women occurs increasingly with age.

DXA-derived total body fat mass (TBFat), total body lean mass (TBLean), and appendicular skeletalmusclemass index (ASMMI) measures reflect the percentage of total body fat (PBF), muscle mass and muscle strength providing a reliable measure for assessment of sarcopenia and obesity [19].

For instance, among subjects with sarcopenic obesity, we found women suffering of normal weight obese (NWO) syndrome, characterized by normal body weight and BMI, but high TBFat accumulation (PBF ≥ 30%) accompanied by TBLean mass deficiency [20].

The key problem for this disease is diagnosis. In order to avoid the risk of sarcopenia in the elderly and to prevent it in the young population, the diagnosis of sarcopenia requires the utilization of various methods, including body composition evaluation and metabolic, functional, and genetic approach. The assessment of the physical status in association with genotype represents very important information to evaluate both the health status and the quality of life. Moreover, while a number of risk factors and diagnostic methodologies are available, it would be very useful to be able to develop additional predictive tools and risk indexes for this pathology.

Given the clinical significance of sarcopenia and the involvement of TP53 in muscle differentiation and homeostasis, the purpose of the present study was to investigate the allelic frequency of the TP53 codon 72 in exon 4 polymorphism and to determine whether this polymorphism is associated with ASMMI in normal weight population (NW with a PBF < 30%), NWO, and preobese-obese (Preob-Ob) subjects, with PBF ≥ 30% [20–22].

We comprehensively analysed anthropometric values, TBFat, and TBLean in a TP53 polymorphism association study. Moreover, we test the possibility to define a function for predicting the risk of sarcopenia due to the simultaneous presence of obesity and TP53 gene polymorphism and provide a simple online tool to apply these calculations.

## 2. Methods

### 2.1. Study Design and Subjects

The study was conducted on a sample of 140 Italian Caucasian women (aged 18 to 65 years), recruited from ongoing studies at the Division of Clinical Nutrition and Nutrigenomics, Department of Biomedicine and Prevention, University of Rome “Tor Vergata” (Italy), from May 2013 to February 2014. A completed screening of anthropometry and body composition was assessed using standardized equipment.

Participation in the study included a complete medical history to gather information about health status, current medications including supplements of vitamin and mineral, alcohol drinking, smoking, dietary intake, and physical activity (PA). Subjects with acute diseases, severe liver, heart, or kidney dysfunctions, endocrine disorders (diabetes, hypothyroidism, or hyperthyroidism), cancer, or other conditions capable of altering body composition (AIDS, Paget's, gastroenteropathies with malabsorption, neuromuscular diseases, rheumatic diseases, mild to severe cognitive impairment, or disability) were excluded. The use of certain drugs (steroids, diuretics) was also a reason for exclusion. Moreover, individuals that either smoke or take medications were excluded from the study. A complete assessment of anthropometry and body composition was performed.

The subjects were categorized according to PBF cutoff point [23] in (1) women having a PBF < 30% and (2) women with a PBF ≥ 30%.

Moreover, we categorized these groups also according to BMI (<25 kg/m^2^ and ≥25 kg/m^2^), into three groups: (1) NW women having a BMI < 25 kg/m^2^ and PBF < 30%; (2) women with PBF ≥ 30% including NWO women with BMI < 25 kg/m^2^ and PBF ≥ 30% [20] and (3) Preob-Ob women with a BMI ≥ 25 kg/m^2^ 10 and PBF ≥ 30% [24].

Study design was clearly written in lay-person language and provided to each study subject. A written informed consent was obtained from each patient and the study protocol conforms to the ethical guidelines of the Declaration of Helsinki.

This trial is registered with ClinicalTrials.gov NCT01890070.

### 2.2. Anthropometric Measurements

Anthropometric parameters for all participants were performed according to standard methods by trained personnel (body weight, height, and hip and waist circumferences), with participant wearing only light underwear and without shoes. After a 12-hour overnight fast, all subjects underwent anthropometric evaluation. Anthropometric measurements for all participants according to standard methods were carried out [22]. All the individuals were instructed to take off their clothes and shoes before undergoing the measurements.

Waist and hip measures were taken using a flexible steel metric tape to the nearest 0.5 cm, with subjects standing with arms relaxed by their side and balanced on both feet. The tape was held tight to the skin but without compression of tissue. Hip circumference was also measured according to International Society for the Advancement of Kinanthropometry protocol [24], taken at the greatest posterior protuberance of the buttocks. Waist circumference (WC) was measured just above the iliac crest as recommended in the National Institute of Health Guidelines [25].

Body weight (kg) was measured to the nearest 0.1 kg, using a balance scale (Invernizzi, Rome, Italy). Height (m) was measured using a stadiometer to the nearest 0.1 cm (Invernizzi, Rome, Italy).

Body mass index (BMI) was calculated using the formula: BMI = body weight/height^2^ (kg/m^2^).

### 2.3. Dual X-Ray Absorptiometry (DXA)

The total body composition was assessed by DXA (iDXA, G.E. Medical Systems, WI, USA), according to the previously described procedure [24].

The technique combined a total body scanner, an X-ray source, an internal wheel to calibrate the bone mineral compartment, and an external lucite/aluminium phantom to calibrate the fat compartment. Standard DXA quality control and calibration measures were performed prior to each testing session. The subjects were instructed not to exercise within 24 h from the test. The subjects were given complete instructions on the testing procedure. Individuals were asked to remove all clothing except for undergarments including shoes, socks, and metal items prior to being positioned on the DXA table. Scans were performed with individuals in a supine position. The entire body was scanned beginning from the top of the head and moving in a rectilinear pattern down the body to the feet. The average measurement time was 20 min. The effective radiation dose from this procedure is about 0.01 mSv. The coefficient of variation (coefficient of variation = 100 × SD/mean) intra- and intersubjects ranged from 1% to 5%. The coefficient of variation for bone measurements is less than 1%; coefficient of variation on this instrument for five subjects scanned six times over a nine-month period was 2.2% for TBFat and 1.1% for TBLean; total PBF was calculated as TBFat mass divided by total mass of tissues (TBFat + TBLean + TBBone) × 100.

Resting metabolic rate (RMR) was calculated according to the previously described procedure [20].

ASMMI was calculated as follow = appendicular skeletal muscle mass (kg)/height (m)^2^. Female subject was defined sarcopenic with a relative ASMMI < 5,47 kg/m^2^ [19].

### 2.4. Genotyping

p53 codon 72 genotype was determined according to the method of de La Calle-Martin [26].

*PCR Primers*. The primer sequences corresponded to the 4th exon of the human p53 gene.

Sense oligo 5′-AATGGATGATTTGATGCTGTCCC-3′.

Antisense oligo 5′-CGTGCAAGTCACAGACTTGGC-3′.

*Polymorphism*. AccH (CGCG) digest of the amplified fragment identifies two alleles: Al = 259 bp and A2 = 160 bp + 99 bp.

*Chromosomal Localization*. The polymorphic AccH recognition site occurs within the 4th exon of the human p53 locus (17ql3).

*PCR Conditions*. PCRs were carried out in a total volume of 250 *μ*L containing 500 ng of genomic DNA, 50 pmoles of each primer, 2 mM MgCl_2_, 200 AM dNTPs, 50 mM KCl, 20 mM Tris-pH 8.3, and 0.1% gelatine. The amplification was performed for 35 cycles with an annealing temperature of 62°C. The amplified DNA was digested overnight with a tenfold excess of AccH. DNA fragments were resolved by electrophoresis through a 2% agarose gel and analyzed under a UV source, using the Molecular Imager Gel Doc image analysis system.

### 2.5. Statistical Analysis

Data are presented as group median ± standard error (SE) or percentage.

The nonparametric Mann-Whitney test was used when comparing two groups. A Pearson's simple correlation was used to study the association between 2 variables. Data were analyzed to check assumptions about the distribution of the measured variables. Three genotype groups were first considered to check differences in considered variables between groups. Because a dominant or recessive effect existed, analysis was repeated comparing *Arg/*Arg genotype versus carriers of *proallele groups. A *χ* ^2^ test was also used to evaluate the Hardy-Weinberg equilibrium of the observed genotype frequencies with respect to the general population.

We further calculated odds ratios (OR) and relative risk (RR) with 95% confidence intervals (CI) for sarcopenia-risk factors with a binary logistic regression model. Population attributable risks (PAR), expressed as percent in the text, were computed using the method described by Bruzzi et al. [27], which allows to be estimated on the basis of data from case-control studies; these risks are expressed as percent in the text. The method requires the knowledge of the OR and of the distribution of risk exposure only among case subjects, assuming that they represent the whole case population.

Statistical analysis, OR, and their confidence intervals (CI) were performed using the computer software package SPSS for Windows, version 13.0; SPSS, Chicago, IL. The logistic response model was used to predict the probability associated with each value of the binary response [28]. A stepwise procedure was used to select the most important predictors [29]. Multivariable logistic regression model was built for sarcopenia-risk assessment. Age and PBF were used as nongenetic effects in the model. The final model was analysed using the Statistical Analysis System, SAS Institute Inc., version 9.3. The level of significance was fixed at *P* ≤ 0.05 for all the procedures.

The web calculator was designed with standard html, CSS, and JavaScript for the user interface and PHP for data processing.

## 3. Results

One hundred forty Caucasian Italian women gave informed consent and were enrolled: 127 women were eligible for the study design according to inclusion criteria. The anthropometric, body composition, and biochemical parameters in the study group were shown in Table 1.

Characterization of the nutritional status by BMI revealed that 0.79% (*n* = 1) were underweight, 44.88% (*n* = 57) were normal weight (eutrophic), 19.69% (*n* = 25) were overweight, and 34.64% (*n* = 44) were obese. Moreover 4.54% of subjects classified as obese by BMI were sarcopenic.

According to PBF cutoff classification, 14.17% of total women were NW and 85.16% were obese (35.8% NWO and 64.2% Preob-Ob, resp.).

Moreover, according to attributable risk (PAR) we found that up to 21% of sarcopenia incidence was dependent on obesity (PAR = 0.21; 95% CI = 0.08–0.55, *P* = 0.0009).

No significant differences in W/H ratio and lean arms were observed between the NW and NWO women. BMI, WC, PBF, TBF, and lean legs were significantly different between NW, NWO, and Preob-Ob (*P* < 0.05). As expected, all parameters of body composition were significantly different between the Preob-Ob and NW groups (*P* < 0.05). Preob-Ob women showed higher amount of fat mass, evaluated as PBF and TBFat (*P* < 0.05) with NW and Preob-Ob women (*P* < 0.05).

No significant differences of ASMMI were observed between the NW and NWO women. Moreover, Preob-Ob subjects showed higher ASMMI with respect to other groups (*P* < 0.05).

The characteristics of subjects, classified according to their genotypes, were shown in Table 2. All subjects were successfully genotyped for the *Arg/*Pro p53 codon 72 polymorphism, distinguishing in wild type homozygous (Arg/Arg), heterozygous (Arg/Pro), and mutant homozygous (Pro/Pro) genotypes.

A total of 67 women (52.72%) had the Arg/Arg genotype with an average age of 37, 64 ± 13, 64 years, 54 women (42.52%) had the Arg/Pro genotype with an average age of 35, 42 ± 11, 73 years, and 6 women had the Pro/Pro genotype with an average age of 37, 82 ± 10, 49 years.

No statistical difference in the mean age was observed.

In a second step statistical analysis was performed on the study population divided into 2 subgroups, *Arg/*Arg genotype and carriers of *proallele TP53 codon 72 polymorphism (Arg/Pro + Pro/Pro).

In NW subjects, TBL and RMR between *Arg/*Arg genotype and carriers of *proallele were significantly different (*P* < 0.05).

Among overall *Argo/*Arg genotype, NWO showed significant differences in PBF, TBFat, lean legs, TBLean, and RMR with respect to NW (*P* < 0.05). Preob-Ob showed significant differences in BMI, WC, HC, and W/H values with respect to NW and NWO (*P* < 0.05).

Among overall carriers of *proallele, NWO showed significant differences in BMI, WC, HC, PBF, TBFat, and lean arms values with respect to NW (*P* < 0.05). Moreover, significant differences were observed in W/H, lean legs, TBLean, and RMR with respect to Preob-Ob (*P* < 0.05).

In NW subjects ASMMI between *Arg/*Arg genotype and carriers of *proallele were significantly different (*P* < 0.05). Moreover, significant differences were observed in ASMMI values between NW and Preob-Ob (*P* < 0.05).

According to ASMMI values, 38.89% of NW (28.6% *Arg/*Arg genotype and 71.4% carriers of *proallele) and 33.33% of NWO (61.5% *Arg/*Arg genotype and 38.5% carriers of *proallele) were sarcopenic. Moreover, 4.29% of Preob-Ob were sarcopenic and all were *Arg/*Arg genotype.

By dividing the women population into sarcopenic and nonsarcopenic individuals, it was observed that *Arg/*Arg genotype increases sarcopenia risk to 20% (*Arg/*Arg genotype OR = 1.20; 95% CI = 0.48–2.9; *proallele carriers OR = 0.83; 95% CI = 0.83–2.06).

The risk of being sarcopenic for *Arg/*Arg genotype women, with a PBF ≥ 30%, is 31% higher than women carriers of *proallele with a PBF < 30% (RR = 0,31, 95% CI = 0,15–0,66, *P* = 0,0079).

Furthermore, in this investigation the probability of sarcopenia was modeled; *P* represented the probability that the event of sarcopenia will occur, with a number between 0 and 1, according to TP53 gene polymorphism, age, and PBF:

(1) P(X,Y,Z)=ln⁡π(X,Y,Z)(1−(X,Y,Z))=−2,4030−0,0379∗(X1−X3)+1,0018∗(X2−X3)+0,017∗Y+0,0728∗Zπ(X,Y,Z)=eP(X,Y,Z)/(1+e(X,Y,Z)).

The variable *X* = (*X*_1_, *X*_2_, *X*_3_) indicates the presence or absence of the TP53 gene polymorphism. In the case of the Arg/Arg genotype the value of *X*_1_ = 1 and *X*_2_ = *X*_3_ = 0. In the case of Arg/Pro genotype the value of *X*_2_ = 1, and *X*_1_ = *X*_3_ = 0. In the case of Pro/Pro genotype the value of *X*_3_ = 1 and *X*_1_ = *X*_2_ = 0. The variable *Y* is the age of the subject, and *Z* is the PBF.

The *P* response variable is as follows: *P* = 0 means no risk; *P* = 1 means 100% probability of sarcopenia risk.

This equation allows the prediction of individual sarcopenia risk on the base of PBF, age, and TP53 gene polymorphism. We have developed an online tool to apply this equation that is available for public use at http://immuno.bio.uniroma2.it/sarcopenia-p53-calculator/.

## 4. Discussion

In the last years, several genetic studies were performed in order to discover genes involved in the development of obesity susceptibility, osteoporosis, and insulin resistance. More than 300 genes have been putatively identified as involved in these processes [26]. Still, the genetic basis of sarcopenia is largely unknown.

Human aging is characterized by increased levels of physical disability due to, at least in part, loss of muscle strength that depends on both decrease in muscle mass and accumulation of intermuscular adipose tissue (IMAT), contributing to the decline of muscle quality, predicting sarcopenia, and increasing risk of mobility impairment [30, 31].

p53 is a tumour suppressor gene which responds to a variety of stress signals, including DNA damage, resulting in cell cycle arrest, apoptosis, or senescence, and several studies suggest that p53 protein activity plays a role in regulating muscle homeostasis, with an important role in the process of myoblasts differentiation [32–35]. It is therefore not surprising that the allelic status of p53 might influence myoblasts differentiation, thereby correlating with the risk of developing sarcopenia, as we show in the present paper. In fact, it has been demonstrated that, in the presence of differentiation-promoting stimuli, p53-defective myoblasts exit from the cell cycle but do not differentiate into myocytes and myotubes, thereby implying a role for p53 in skeletal muscle differentiation [36, 37]. Moreover, p53 is involved in the NF-*κ*B and PI3 K/Akt pathways that are central in controlling muscle size, promoting protein synthesis, and blocking degradation [38–41].

Considering the great interest toward p53 function in the context of skeletal muscle differentiation and apoptosis and the established difference in activities of Arg and Pro variants, we investigated the possible relationship between the polymorphism at codon 72 of p53 gene, body composition, and ASMMI in NW, NWO, and Preob-Ob women, to highlight a potential connection of these genetic variants with the predisposition to sarcopenia.

Aims of our work were to identify which group of women, as a function of the weight, body lean, and fat mass, were at increased risk of reduction of skeletal muscle mass that could lead to sarcopenia and to verify if the codon 72 in exon 4 p53 gene polymorphism could be associated with this condition.

Therefore, we used TP53 genotyping as a screening factor for this disease. Finally, we tried to identify a model to predict the risk of sarcopenia, according to age, genotype, and body fat mass. We have initially classified our study population according to BMI and PBF, to identify obese subjects, and to ASMMI, to identify sarcopenic subjects.

In the present study, a significant difference in the diagnosis of obesity between BMI and PBF was observed with an error percentage of 40.68% (*P* < 0.05). According to PBF cutoff classification, only 14.17% of the total were eutrophic and normal weight, while all the others were obese.

Since the NWO women had a higher prevalence of metabolic syndrome (MS), cardiovascular diseases (CVD), high oxidative stress, and low RMR become necessary to evaluate body composition to assess the risk of sarcopenia [42–50]. It was previously described that the prevalence of NWO in the general population varies from 2% to 28% in women and is almost nonexistent in men (less than 3%) [42, 43]. In particular, we observed a frequency of 30.7% of NWO in the general population.

Moreover, in the total population, 5.5% of NW, 10.24% of NWO, and 2.36% of Preob-Ob were sarcopenic according to ASMMI values.

As previously described, BMI does not take into consideration muscle mass loss, exacerbating misclassifications of sarcopenic obesity [51]. In fact, according to BMI, 4.54% of subjects were obese and sarcopenic. However, according to PBF cutoff classification we found that 14.68% of individuals were affected by sarcopenic obesity (81.25% NWO and 18.75% Preob-Ob, resp.). Therefore, when we checked the frequency of sarcopenic obesity, based on body composition in this study, we preferred to group the NWO with the Preob-Ob rather than with the NW, despite their phenotype [24].

Moreover, to assess the relationship between sarcopenia and obesity we calculated the population attributable risk (PAR) and found that up to 20% of sarcopenia incidence could be reduced by appropriate detection and treatment of obesity.

By dividing the study population according to genotype and body composition, significant differences in the three study groups, NW, NWO, and Preob-Ob, and intergenotypes were found. Among the same group NW carriers of *Pro have lower weight with respect to *Arg/*Arg genotype; the same results were observed between Preob-Ob. In NWO no significant differences were observed. Moreover, among the same genotype, NW carriers of *proallele have significantly lower weight with respect to NWO and Preob-Ob; on the contrary Preob-Ob carriers of *proallele are the fattest.

In NWO women carriers of *proallele, higher values in BMI, but not in weight, were observed.

Regarding WC, among the same group, NW carriers of *proallele have lower WC with respect to *Arg/*Arg genotype; on the contrary, in Preob-Ob the lower value was observed in *Arg/*Arg genotype. Among NWO women, carriers of *proallele show the higher value with respect to *Arg/*Arg genotype. WC or waist-to-hip ratio has been used as a proxy measure for body fat distribution when investigating the health risk increased with an increasing ratio. Some studies have suggested that WC, either alone or in combination with BMI, may have a stronger relation to some health outcomes than BMI alone [52]. We considered the results obtained on WC and W/H ratio of great importance, because WC reflects abdominal or intra-abdominal fat, and H reflects different aspects of body composition in the gluteofemoral region, with consequently different physical and metabolic characteristics of these two regions. This may be due to the tendency for abdominal adipocytes to enlarge (hypertrophy), whereas subcutaneous femoral adipocytes increase in number (hyperplasia); moreover, hypertrophic adipocytes tend to be associated with dyslipidemia and insulin resistance [53]. It has been suggested that the composition of gluteal fat deposits corresponds more closely to that of visceral deposits rather than femoral deposits [54].

As reported in Table 2, a significant reduction (*P* < 0.05) of RMR between NWO, NW, and Preob-Ob women was observed, independently of genotype. We confirmed, according to our previous results [44], a lower RMR in NWO with respect to NW and Preob-Ob women. The effects of body composition on RMR in NWO were demonstrated comparing the RMR between NW, Preob-Ob, and NWO; NWO and the NW have a lowest RMR with respect to Preob-Ob (*P* < 0.05). According to genotype, the RMR in Preob-Ob carriers of *proallele is significantly higher than *Arg/*Arg genotype. NW carriers of *proallele have significantly lower values than *Arg/*Arg genotype, tending towards a value similar to NWO. The RMR is the lowest due to a reduction in metabolically active fat free mass. Thus, the measurement of energy expenditure normalized to metabolically active mass should provide a tool to identify hyper- and hypometabolic states at an early stage.

The NWO groups showed the same trend of the Preob-Ob women in terms of fat mass content and of ASMMI levels (*P* > 0.05). In the NW groups we found an opposite trend with respect to the Preob-Ob women. For the first time, we highlighted that the NW *proallele carriers have ASMMI values lower than normal cutoff, indicating a sarcopenic predisposition.

Considering that the PBF > 30% increased risk of sarcopenia and the fact that, between the two genotypes in our sample, genotype *Arg/*Arg is associated with the risk of sarcopenia, *Arg/*Arg subjects with a PBF > 30% appear to be those most at risk of developing the disease (31% higher than the *proallele subjects with PBF < 30%).

In the present study, we developed a logistic model to assess the sarcopenia risk, based on age, genetic analysis, and body composition, that may be applicable to Italian women ranging widely in age. Note that our study cohort was selected from a population of patients and affiliates of our Division of Clinical Nutrition and Nutrigenomic: although it was not taken at random from the Italian population, this sample can be regarded as representative of women at risk and requiring medical examination.

Finally, we mention some limitations of the study. Our cohort is composed of a limited number of subjects and it is population specific. Because the number of included subjects was relatively low, validation of the results in larger, independent cohorts is recommended to confirm our results and exclude the risk of false negative findings.

Some reports suggest an increase of inflammatory catabolic signals with age and a reduction of anabolic signals to muscle present in young adulthood, favoring onset of sarcopenia [55]. However, our study was performed only in adult female subjects, excluding elderlies. We investigate only female population, because the prevalence of NWO syndrome and sarcopenic obesity is higher in women than in men [56].

Furthermore, we were unable to perform laboratory evaluations for all participants, and we lack muscle function data (strength or performance), thereby sometimes hindering a complete sarcopenia diagnosis.

However, our results support the hypothesis that codon 72 p53 gene polymorphism could be associated to sarcopenia, in a way that is independent of BMI.

Moreover, physicians should take full advantage of the predictive model and simple equation generated by the present study for the assessment of sarcopenia risk. For this reason, we have developed an online tool that provides free access to the calculations for this equation, now accessible at http://immuno.bio.uniroma2.it/sarcopenia-p53-calculator/. The PBF can be either inputted directly or calculated from height, weight, waist circumference, and hip circumference as described in our previous study [57].

To prevent and manage poor quality of life related to sarcopenia, greater attention and early evaluation should be given to subjects showing a lower ASMMI.

The changes in medicine are represented by a new systems approach to studying, understanding, and monitoring fundamental biological and disease processes that will trigger the emergence of personalized medicine focused on the integrated diagnosis and treatment and prevention of disease in individual patients [58].

The key elements of all risk-prediction tools, from baseline risk assessment to analysis of appropriate therapeutics, will benefit from the molecular understanding of the pathogenesis of disease, along with the identification of predictive factors, particularly biomarkers that anticipate or quantify the pathogenic process. Moreover, an integrated approach and the development of new mathematical and computational methods, for extracting maximum information from molecular and genetic information on individuals and from other clinical data and history, as well as integrating all of this information into predictive models, become necessary for the application of the P4 medicine [58, 59].

Since chronic diseases, such as sarcopenia, develop as a consequence of an individual's baseline susceptibility coupled with exposure to environmental factors, including life style, diet, and body composition, personalized risk prediction, and strategic health-care planning will facilitate the “prospective health care,” according to predictive, preventive, and personalized medicine [56]. The prevention, early diagnosis, and therapy of malnutrition represent a great clinical importance, particularly to preserve physical functional capacity and thus quality of life in elderly and young women.

In conclusion, this study shows that a genetic approach, like genotyping of codon 72 p53 polymorphism, associated with body composition evaluation is highly desirable, in order to prevent and manage the risk of muscle mass loss or ASMMI reduction in women population.

The developed equation for the calculation of the sarcopenia risk can be accessed online at the following address: http://immuno.bio.uniroma2.it/sarcopenia-p53-calculator/.

## Figures and Tables

**Table 1:** Anthropometric and body composition (DXA) parameters in study groups.

Parameters	Min–Max	Mean ± SD
Age (y)	18,00–65,00	34,50 ± 1,12
Height (cm)	148,00–179,00	161,50 ± 0,48
Weight (Kg)	46,20–147,40	68,10 ± 2,07
BMI (Kg/m^2^)	17,13–57,00	26,00 ± 0,80
WC (cm)	61,00–137,00	78,50 ± 1,72
HC (cm)	64,00–150,00	103,00 ± 1,45
W/H	0,67–1,16	0,76 ± 0,01
PBF (%)	20,30–58,10	41,40 ± 0,82
TBF (Kg)	6,20–80,06	27,59 ± 1,42
Lean arms (Kg)	0,35–8,82	3,74 ± 0,10
Lean legs (Kg)	0,97–22,62	13,06 ± 0,24
TBL (Kg)	28,54–65,21	38,12 ± 0,62
RMR (Kcal)	1058,00–2417,00	1412,50 ± 23,15
ASMMI (kg/m^2^)	1,89–10,81	6,35 ± 0,11

BMI: body mass index; WC: waist circumference; HC: hip circumference; W/C: waist-to-hip ratio; PBF: percentage of total body fat mass; TBFat: total body fat mass; TBLean: total body lean mass; RMR: resting metabolic rate; ASMMI: appendicular skeletal muscle mass index.

**Table 2:** Anthropometric and body composition (DXA) parameters according to p53 codon 72 polymorphism genotypes.

	NWO			NW			Preob-Ob		
	All (*n* = 39)	∗Arg/∗Arg genotype (*n* = 18)	Carriers of ∗proallele (*n* = 21)	All (*n* = 18)	∗Arg/∗Arg genotype (*n* = 10)	Carriers of ∗proallele (*n* = 8)	All (*n* = 70)	∗Arg/∗Arg genotype (*n* = 39)	Carriers of ∗proallele (*n* = 31)
Height (cm)	161,00 ± 0,88	162,50 ± 0,94	161,00 ± 1,42	165,00 ± 1,10^a^	164,50 ± 1,33	165,25 ± 1,94	161,00 ± 0,64^c^	161,00 ± 0,78^m^	162,00 ± 1,09
Weight (Kg)	58,50 ± 0,86	56,10 ± 1,22	58,80 ± 1,20	54,25 ± 0,99^a^	56,10 ± 1,00	52,00 ± 1,60^e,h^	87,05 ± 2,52^b,c^	84,00 ± 3,06^i,m^	95,00 ± 4,13^l,n^
BMI (Kg/m^2^)	22,24 ± 0,26	21,67 ± 0,40	23,10 ± 0,30^d^	20,60 ± 0,34^a^	21,03 ± 0,27	19,04 ± 0,62^h^	32,98 ± 0,96^b,c^	30,74 ± 1,23^i,m^	33,78 ± 1,50^l,n^
WC (cm)	72,50 ± 1,15	72,00 ± 0,97	73,75 ± 1,88	66,00 ± 1,12^a^	71,00 ± 1,45	65,00 ± 1,60^h^	95,25 ± 2,25^b,c^	94,50 ± 2,68^i,m^	101,00 ± 3,74^l,n^
HC (cm)	97,50 ± 1,03	96,50 ± 1,31	97,50 ± 1,57	91,00 ± 1,85^a^	95,00 ± 1,22	90,50 ± 3,47^h^	115,00 ± 1,79^b,c^	113,50 ± 2,23^i,m^	117,00 ± 2,92^l,n^
W/H	0,73 ± 0,01	0,72 ± 0,01	0,76 ± 0,01^d^	0,74 ± 0,02	0,74 ± 0,01	0,73 ± 0,04	0,82 ± 0,01^b,c^	0,81 ± 0,01^i,m^	0,83 ± 0,02^l,n^
PBF (%)	34,40 ± 0,62	33,35 ± 1,02	35,60 ± 0,78	25,10 ± 0,75^a^	26,00 ± 0,84^g^	24,10 ± 1,28^h^	47,80 ± 0,64^b,c^	46,20 ± 0,84^i,m^	48,60 ± 1,00^l,n^
TBF (Kg)	20,02 ± 0,82	19,06 ± 1,20	21,37 ± 1,12	13,65 ± 0,60^a^	14,32 ± 0,62^g^	11,82 ± 1,04^h^	38,07 ± 1,71^b,c^	36,73 ± 2,04^i,m^	44,04 ± 2,89^l,n^
Lean arms (Kg)	3,43 ± 0,13	3,42 ± 0,23	3,60 ± 0,13	2,68 ± 0,22	3,81 ± 0,28	2,07 ± 0,29^h^	4,07 ± 0,15^b,c^	3,93 ± 0,20^i^	4,36 ± 0,21^l,n^
Lean legs (Kg)	11,91 ± 0,35	11,79 ± 0,27	12,07 ± 0,61	12,82 ± 0,25^a^	13,07 ± 0,36^g^	12,30 ± 0,32	14,63 ± 0,34^b,c^	14,11 ± 0,47^i^	14,99 ± 0,45^l,n^
TBL (Kg)	34,92 ± 0,48	34,79 ± 0,63	34,92 ± 0,73	37,81 ± 0,58^a^	38,15 ± 0,73^g^	36,56 ± 0,77^e^	43,32 ± 0,90^b,c^	42,33 ± 1,09^i^	45,67 ± 1,48^l,n^
RMR (Kcal)	1294,00 ± 17,83	1289,00 ± 23,20	1294,00 ± 27,01	1401,00 ± 21,58^a^	1413,50 ± 27,20^g^	1355,00 ± 28,33^e^	1605,00 ± 33,42^b,c^	1569,00 ± 40,37^i^	1692,00 ± 54,97^l,n^
ASMMI (kg/m^2^)	5,93 ± 0,14	5,83 ± 0,15	5,94 ± 0,24	5,79 ± 0,13	5,94 ± 0,16	5,38 ± 0,17^e^	7,10 ± 0,15^b,c^	6,96 ± 0,22^i,m^	7,30 ± 0,19^f,n^

BMI: body mass index; WC: waist circumference; HC: hip circumference; PBF: percentage of total body fat mass; TBFat: total body fat mass; TBLean: total body lean mass; RMR: resting metabolic rate; ASMMI: appendicular skeletal muscle mass index.

^a^ *P* ≤ 0,05 NWO all versus NW all; ^b^ *P* ≤ 0,05 NWO all versus Preob-Ob all; ^c^ *P* ≤ 0,05 NW all versus Preob-Ob all; ^d^ *P* ≤ 0,05 NWO ∗Arg/∗Arg genotype versus NWO carriers of ∗proallele; ^e^ *P* ≤ 0,05 NW ∗Arg/∗Arg genotype versus NW carriers of ∗proallele; ^f^ *P* ≤ 0,05 Preob-Ob ∗Arg/∗Arg genotype versus Preob-Ob carriers of ∗proallele; ^g^ *P* ≤ 0,05 NWO C∗Arg/∗Arg genotype versus NW ∗Arg/∗Arg genotype; ^h^ *P* ≤ 0,05 NWO carriers of ∗proallele versus NW carriers of ∗proallele; ^i^ *P* ≤ 0,05 NWO C∗Arg/∗Arg genotype versus Preob-Ob ∗Arg/∗Arg genotype; ^l^ *P* ≤ 0,05 NWO carriers of ∗proallele versus Preob-Ob carriers of ∗proallele; ^m^ *P* ≤ 0,05 NW ∗Arg/∗Arg genotype versus Preob-Ob ∗Arg/∗Arg genotype; ^n^ *P* ≤ 0,05 NW carriers of ∗proallele versus Preob-Ob carriers of ∗proallele.
